# Equity in family planning policies and programs in Uganda: conceptualization, dimensions and implementation constraints

**DOI:** 10.1186/s12939-024-02143-1

**Published:** 2024-03-11

**Authors:** Noel Namuhani, Rhoda K. Wanyenze, Suzanne N. Kiwanuka, Joseph K. B. Matovu, Fredrick E. Makumbi

**Affiliations:** 1 Department of Health Policy Planning and Management , Makerere University School of Public Health, P. BOX 7072, Kampala, Uganda; 2 Department of Disease Control and Environmental Health, Makerere University School of Public Health, Kampala, Uganda; 3 Department of Community and Public Health, Busitema University Faculty of Health Sciences, Mbale, Uganda; 4 Department of Epidemiology and Biostatistics, Makerere University School of Public Health, Kampala, Uganda

**Keywords:** Equity in family planning, Dimensions of equity, Definitions of equity, Equity implementation constraints

## Abstract

**Background:**

Equity is at the core and a fundamental principle of achieving the family planning (FP) 2030 Agenda. However, the conceptualization, definition, and measurement of equity remain inconsistent and unclear in many FP programs and policies. This paper aims to document the conceptualization, dimensions and implementation constraints of equity in FP policies and programs in Uganda.

**Methods:**

A review of Ugandan literature and key informant interviews with 25 key stakeholders on equity in FP was undertaken between April and July 2020. We searched Google, Google Scholar and PubMed for published and grey literature from Uganda on equity in FP. A total of 112 documents were identified, 25 met the inclusion criteria and were reviewed. Data from the selected documents were extracted into a Google master matrix in MS Excel. Data analysis was done across the thematic areas by collating similar information. Data were analyzed using thematic content analysis approach.

**Results:**

A limited number of documents had an explicit definition of equity, which varied across documents and stakeholders. The definitions revolved around universal access to FP information and services. There was a limited focus on equity in FP programs in Uganda. The dimensions most commonly used to assess equity were either geographical location, or socio-demographics, or wealth quintile. Almost all the key informants noted that equity is a very important element, which needs to be part of FP programming. However, implementation constraints (e.g. lack of quality comprehensive FP services, duplicated FP programs and a generic design of FP programs with limited targeting of the underserved populations) continue to hinder effective implementation of equitable FP programs in Uganda. Clients’ constraints (e.g. limited contraceptive information) and policy constraints (inadequate focus on equity in policy documents) also remain key challenges.

**Conclusions:**

There is lack of a common understanding and definition of equity in FP programs in Uganda. There is need to build consensus on the definitions and measurements of equity with a multidimensional lens to inform clear policy and programming focus on equity in FP programs and outcomes. To improve equitable access to and use of FP services, attention must be paid to addressing implementation, client and policy constraints.

## Background

Family planning (FP) is one of the most cost-effective health and development investments for countries. It improves the health of mothers, children, and families, boosts economies and empowers women [1]. Worldwide, FP has resulted in reductions in maternal and infant mortality and other adverse outcomes [2, 3]. However, significant inequities exist across regions and countries and within countries. The levels of unmet need for FP and low contraceptive use remain a big challenge in many low income countries [4]. Sub-Saharan Africa (SSA) has the highest number of women who have an unmet need for contraception globally. One in four women of reproductive age in East Africa have an unmet need for FP, with Uganda having the highest unmet need [5].

Although there has been a significant decline in the unmet need for FP for women from 34 to 26% in Uganda [6], achieving the national costed FP implementation plan (CIP) target of 10%, remains a challenge. Furthermore, variations in FP indicators exist between sub-regions, age groups and education levels [7]. According to the 2016 Uganda demographic Health Survey (UDHS), use of modern contraception was higher among the educated, and women in the highest wealth quintiles, and was disproportionately higher in the urban (41%) than rural areas (33%), with contraceptive prevalence rate (CPR) being lowest in Karamoja (7%) and highest in Bugisu (43%) and Kigezi (43%). Unmet need for FP also varied by women’s wealth-quintile; lower in the lowest quintile and highest in the wealthiest quintile, while the average children ever born (CEB) was higher in the rural relative to the urban areas [6].

Inequities in FP service access and utilization have adverse outcomes for the unreached women, children, and their communities [8]. Poorer health outcomes such as infant low birth weight, infant mortality, and maternal mortality and morbidity [9, 10] as well as the increased risks of unintended birth especially among young/adolescent mothers [11]. It is therefore critical to recognize these inequities and work towards further understanding and addressing their causes so as to enable implementation of appropriate targeted interventions to minimize or eliminate these disparities.

Equity is at the core and a fundamental principle to achieving the FP2030 targets, where each person has the same right and access to quality FP, regardless of their geography, socioeconomic status, gender, or culture [12]. Equity in health refers to the absence of unfair, avoidable and remediable differences in health status among groups of people [13, 14]. Equity in FP implies that all people (regardless of their social, economic and geographical background) should have an equal/fair opportunity to access quality FP services and that there are no differences in how they are treated by providers. However, there are discrepancies in the way equity is defined, measured and assessed in many programs and policy documents. In FP programs, equity is interchangeably used with equality, yet there are differences between inequality and inequity [15, 16]. Equality means that the access to services is even across all groups while equity, the access to services is according to need [17].

A number of frameworks (including the US healthy people 2020 framework, human rights framework, *PROGRESS* framework, WHO’s Priority Public Health Conditions Analytic Framework and equity Framework) have been suggested for conceptualizing, defining, operationalizing, programming and identifying dimensions of equity [18–20].

**Fig. 1:**
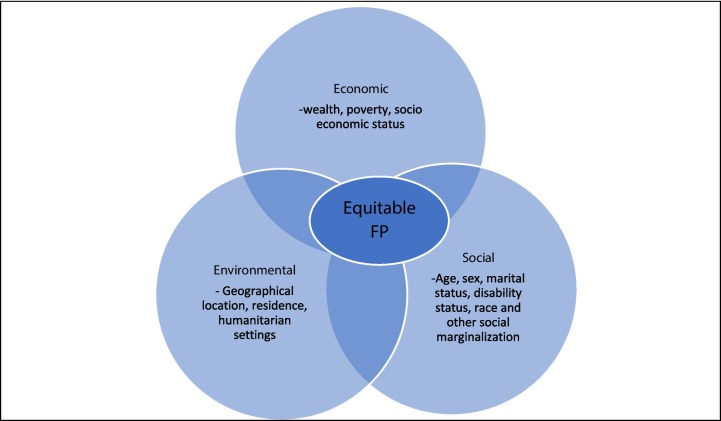
Dimensions of equity in FP (adopted from the US healthy people 2020 framework [21]

The US healthy people 2020 framework, originates from the Healthy People 2020 initiative in the United States, whose one of the primary goals was to achieve health equity, eliminate disparities, and improve the health of all groups [21]. Under this framework, inequities are defined as health differences that are closely linked with economic, social, or environmental disadvantage [19]. The framework categorizes the dimensions of equity into 1) Economic (wealth, poverty, socio economic status), 2) Social (Age, sex, marital status, disability status, race and other social marginalization) and 3) Environmental dimension (geographical location, residence, humanitarian settings) [19]. This framework has been adapted for this study because it categorizes all the dimensions of equity and includes relevant methods for measuring outcomes of interventions to reduce inequities (Fig. 1). The framework informed the themes of inquiry and synthesis of the findings on dimensions for measuring and assessing equity.

The general understanding of how equity is defined and measured in many sexual and reproductive health programs and policies in Uganda has not been explored. This paper documents how equity in FP is defined, assessed and measured in Uganda, and the constraints of achieving equity in FP service delivery. This will inform the ongoing discussions on how equity should be defined and measured. It will contribute to building consensus on the common definition of equity which is important for designing policies and programs with equity as focus and also guide implementation of equitable FP. It will also guide improvements, adjustments and support equitable access to and use of quality, comprehensive FP information and services as well as outcomes for the individuals and couples that are in need.

## Methods

This paper is based on a country consultation to develop a FP research and learning agenda for Uganda, which involved desk reviews, key informant interviews and wide stakeholder consultative workshops to prioritize FP evidence gaps for Uganda across several thematic areas, including equity [22]. The literature review and key informant interviews were conducted to explore how equity in FP programs is defined, measured and to identify constraints of achieving equity in FP programs. The pre-determined themes of inquiry included: conceptualization of equity in FP programs and policies, measurement and assessment of equity, Constraints to achieving equity in FP programs and policies. These were informed by the study objectives. The subthemes for measurement and assessment of equity were informed by the healthy people 2020 framework which categorizes dimensions into 3 major subthemes of social, economic and environmental dimensions. The subthemes for constraints (Implementation, client and policy constraints) emerged from the study findings.

### Literature review

**Fig. 2:**
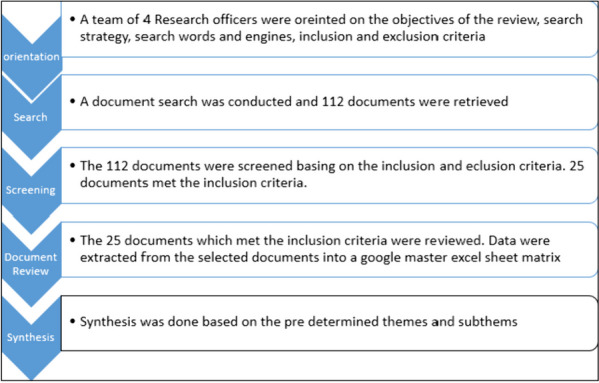
Summary of Document review Process

A review of literature on equity in FP in Uganda was undertaken between April and May 2020, as summarized in Fig. 2. We searched Google, Google Scholar and PubMed for documents that reported on equity in family planning programs or research using the following search terms: “family planning”, “contraceptives”, “equity”, “inequity”, “inequality”, “equality”, “marginalized populations”, “underserved populations”, and “access to FP”. We also checked the websites of FP-focused organizations such as United Nations Populations Fund (UNFPA), Ministry of Health (MOH) and United States Aids Agency for Development (USAID) for additional documents. The documents, which fulfilled the inclusion criteria, were reviewed.

### Inclusion criteria

The criteria used for screening documents included that they be Ugandan documents, with elements of equity, equality, inequity and inequality in FP, and published not more than 10 years prior to 2020. Research articles with national or sub regional data were included while research articles that covered a geographical area of a sub county were excluded from the review.

A total of 112 FP documents were retrieved and screened for inclusion criteria—25 documents satisfied the inclusion criteria and were reviewed. These included FP policy documents such as the costed FP implementation plan, Uganda demographic health survey reports, Performance Monitoring for Action (PMA) reports, national and health sector development plans, research and program reports, program, policy or research briefs, fact sheets, and journal articles. Data were extracted from the selected documents into a Google master excel sheet matrix by trained research officers. There were pre-determined themes of inquiry in the data extraction sheet. The data extraction matrix captured variables such as type and author of document, equity mentioned and defined in the document, equity dimensions addressed in the document, FP outcomes used to assess equity, constraints of achieving equity and policy, program and Research gaps.

### Key informant interviews

To supplement on the literature review, 25 key informant interviews were conducted with key FP stakeholders including policymakers, donors, and program implementers, to explore opinions about the focus of equity in FP programs, equity definitions, measurements, constraints and gaps. These interviews also covered multiple topics including young people, high impact practices and self-care. However, these are not covered in this paper. The key informants were purposively selected based on their expertise, knowledge and contributions to FP programs in Uganda. The sample size was based on feasibility in both time and budget. Due to COVID-19 disruptions, the interviews were conducted virtually via phone or zoom calls using a key informant interview guide, which was developed around the themes of the concept of equity in FP, assessing equity in FP programs and constraints. The interviewers were trained in qualitative interview skills and research ethics prior to conducting the interviews. The interview notes and recordings, were reviewed but were not transcribed directly due to time constraints. However, detailed notes of the interviews were written. A quality control team reviewed the notes and listened to the recording to ensure the summary adequately reflects the main themes of the conversation. The team later analyzed the notes thematically by identifying common subthemes across the interviews, which were integrated into the findings of the desk review. The dimensions and measurement of equity were summarized according to US healthy people 2020 framework [19]. The stakeholders were engaged in study design, implementation and dissemination of study findings.

## Results

The results presented in this section are derived from both the literature review and the key informant interviews.

### A description of documents reviewed and key informants

Twenty-five FP country documents were reviewed and 25 key informants were interviewed (Table 1). The stakeholders consulted included FP implementing partners in the country, Ministry of Health officials, and district health officers at sub national level, civil society organization and FP researchers.

**Table 1:** Background characteristics of the documents and key informant interviews

Variable	Frequency (*N*)
**Types of Documents reviewed**	25
Program documents	7
Research articles	7
Policy documents	3
Issue/policy briefs	3
Working papers	2
Others (book chapter, block, fact sheet)	3
**Key informants**	
**Sex**	
Male	10
Female	15
**Education level**	
Master's degree and above	22
Undergraduate degree and below	3

### Conceptualization of equity in FP programs and policies

In the literature, a limited number (12/25) of documents had an explicit definition of equity (Table 2). But even then, the definitions varied across documents [23–34]. The definitions of equity in policy documents revolved around universal access to FP information and services, as indicated below from some of the documents;

*“Individuals have the ability to access quality, comprehensive contraceptive information and services free from discrimination, coercion and violence”* [35]

*“Achieving universal access to sexual and reproductive health care services, including family planning, information and education, and the integration of reproductive health into national strategies and programs"* [36]

**Table 2:** Summary of documents reviewed, how equity was defined, the dimensions and outcomes used to measure equity

Author and date	Equity definitions	Dimension	Measurement Outcomes
Shannon et al., 2020	Elimination discrimination against women and girls, eradicate violence in both public and private spheres and achieve universal access to sexual and reproductive health (SRH) services	Social, environmental	Method choice
Linnea, 2016	Differences in accessing FP based on: living in urban or rural areas, education, gender, ethnicity, religious affiliation, and income	None	Unmet need, Access to contraceptive information, social acceptance of contraceptives
Clark and Goodhart, 2016	None	None	None
Wright et al., 2017	Individuals have the ability to access quality, comprehensive contraceptive information and services free from discrimination, coercion and violence	Social, other	Access to quality FP
Guttmacher Institute, 2017	None	Social, economic, Environmental	Unmet need for FP, Un intended pregnancies, Access to providers
Bellows et al., 2017	Expanding FP access to the disadvantaged populations	Social, economic, Environmental	Contraceptive use, Un intended pregnancies
USAID, 2016	None	Social, economic, Environmental	Fertility desires, contraceptive use
Koseki and Klein, 2018	Access to FP to marginalized rural and poor populations	Economic, Environmental	Unmet need for family planning, FP method use by choice
Track20, 2015	None	Other	Modern contraceptive use
PMA2020, 2014	None	Social, economic and Environmental	Demand satisfied, Un met need, Contraceptive method mix, Contraceptive prevalence rate(CPR) Unintended birth FP method by choice, Total fertility rate(TFR), Access to information, Satisfaction with provider
Creanga et al., 2011	Inequity exists when people are unfairly deprived of something they want or require to protect them from an unwanted or undesirable condition	Economic	Met Need for FP
Namasivayam et al., 2019	None	Social, Economic	Contraceptive use
UNFPA, 2017	Universal access to sexual and reproductive health services	Social, Economic, and Environmental	Teenage child bearing, CPR
UNFPA, 2015	Implicitly as geographical disparities in CPR and high unmet need for FP among young people	Social, Environmental	CPR, Unmet need for FP
UNFPA, 2017	Implied as expanding access to FP in remote and hard to reach areas	Social and Environmental	Unmet need for FP, TFR, Access to FP
USAID, 2018	Directing FP interventions where they are needed most	Social, Environmental	TFR, Teenage pregnancy
UBOS and ICF, 2018	None	Social, Environmental and Economic	Use of contraception, Unmet need for FP, Contact with FP providers, Decision making about FP, Informed choice, TFR, Teenage pregnancy, Desire for a child/to limit birth, Unwanted birth
Kalangwa and Chelimo, et al., 2018	The extent to which different socio-economic strata affect access and use of FP products and services	Social, economic and Environmental	Use of FP, Unmet need for FP
Akol et al., 2014	Access to FP in hard to reach	Environmental	FP Use
MOH, 2014	Access to family planning without discrimination, coercion, or violence	Social, economic and Environmental	Un met need for FP, TFR, mCPR, Access to FP
MOH, 2015	Universal access to sexual and reproductive health care services, including family planning, information and education	None	TFR, CPR, Unmet need for FP
Ssengooba et al., 2017	None	Social, economic and environmental	Use of contraceptives, unmet need for FP, TFR, discontinuation of contraceptives
Partners in Population and Development, 2017	Universal access to reproductive health, including family planning services	Environmental	Unmet need for FP, Total fertility rate, mCPR
NDPII, 2015	Universal access to FP	None	Child bearing, Teenage pregnancy, unmet need for FP, Fertility rate
MOH, 2018	Geographical access to FP interventions	Social and environmental	Contraceptive use

Program documents also had varying definitions of equity, often focusing on one dimension such as wealth or geography as shown below:

*“Parallel disparities in fertility and in contraceptive use found between poor and wealthy women”* [30]

*“Equity includes disparities in the FP indicators between rural and urban”* [37]

*“Inequity refers to differences in accessing FP based on: living in urban or rural areas, education, gender, ethnicity, religious affiliation, and income”* [34]

Most of the key informants highlighted the lack of a common understanding, definition and dimensions of equity. One of the key informants noted;

*“The question is how should equity be defined, measured and assessed? Is it just about who is left behind? Is equity measured right? Should it be expanded? ... we need to define the dimensions of focus and have all FP partners agree on the definition”* (KI-Development Partner).

Most of the key informants interchangeably used equity with equality. Almost all of the key informants acknowledged that equity is a very important element that needs to be part of the FP programming. However, most of the key informants, especially those supporting the implementation of FP programs, noted that equity was not a focus in most of the FP programs and does not factor into design of specific FP interventions.

*“Most FP programs do not focus on equity. The programs are generic without addressing those in most need. The rural are being left out in designing and accessing FP services compared to the urban dwellers”* (KI-National Level)

On the other hand, a few of the key informants especially at national level noted that equity was a big focus in FP programs and the inequities are being addressed through provision of FP in drug shops, using community health workers, subsidies, prioritizing intervention and programs basing on need. It was also noted that the total Market Approach and National Health Insurance were some of the interventions anticipated to reduce inequities.

*“When MoH is doing FP programming, we focus on equity and the ministry is trying to reach the rural areas with FP services using VHTs and drug shops and other measures like national health insurance and total market approach which are in the pipeline”* (KI-National level)

*“Equity is a key focus and there is deliberate effort to subsidize services dependent on the population purchasing power. Also, interventions are selected based on understanding of target population and the need. We are specifically targeting the young, the poor and the disabled and regions of priority such as Karamoja”* (KI-Development Partner)

### Measurement and assessment of equity

#### Dimensions of equity

In the literature reviewed, the majority (19/25) assessed equity basing on the geographical dimension [6, 23–29, 31–34, 37–43], followed by (17/25) socio demographics (age and education level, disability status, ethnicity) [6, 23, 24, 26–29, 31, 33–35, 38–43] and 12/25 documents assessed equity based on the economic dimension [6, 23, 24, 26, 30–34, 39, 40, 42], as summarized in Table 3. Most of the key informants noted that to assess equity, data tended most often to be disaggregated by income/poverty. This was followed by socio demographics (age and education level, disability status), geographical location and residence.

**Table 3:** Summary of dimensions used to assess equity as found in literature review

Dimension	Items/Indicators	Number of documents (*N* = 25)
Geographical	Residence-rural/urban, regions North/Karamoja, Hard to reach/remote, across countries	19
Socio demographics	sex, age, marital status, parity, ethnicity, religion	17
Economic	Wealth quintiles, poor/rich	12
Women empowerment	employed/working vs house wife [23]	3
Others	New/continuing FP user, place of birth (facility/home) [44]	2

#### Multidimensional assessment of equity

Only 9/25 documents addressed equity based on three dimensions of economic, socio demographic and geographical location [6, 23, 24, 26, 33, 34, 39, 40, 42], 2/25 documents considered four dimensions; economic, socio demographic, geographical location and empowerment [33, 34], while the majority 15/25 assessed equity basing on 2 or less dimensions of either geographical or socio demographic. These dimensions cut across policy, program documents and research articles.

#### Outcomes used to measure equity

In the literature reviewed, the most commonly used health outcomes for measuring equity were modern contraceptive prevalence rate (mCPR) reported in 14/25 documents, followed by unmet need for FP (13/25), total fertility rate (9/25), fertility desires (3/25), teenage/adolescent pregnancies/birth (6/25), and unwanted pregnancy (4/25). Other indicators highlighted in the documents used to measure equity included access to FP services and information (6/25), demand satisfied, FP method mix, satisfaction with quality of FP services, informed choice, sex by choice, median age of women at first sex, median age of women at marriage and median age at first use of contraception, which were noted in one document. Similarly, most of the key informants noted that the program indicators used for measuring equity were number of people using FP, number of people accessing FP and availability of FP commodities in health facilities. Other indicators reported were number of couple years of protection and number of health providers trained to offer FP services.

#### Data sources for measuring equity

The most commonly mentioned data source for measuring equity was the Health Management Information Systems (HMIS). Other data sources included surveys such as PMA2020, demographic health surveys, pilot studies, and researchers from MakSPH and FP atlas. However, accessing data to measure equity was also a challenge due to bureaucracies of obtaining some data, poor quality data and lack of data on the very young women (10–14 years) and People living with a disability (PWD) in most datasets.

### Constraints to achieving equity in FP programs and policies

A total of 20/25 documents [23–30, 32–35, 37–45] noted a number of gaps and constraints to achieving equity in FP programs, which can be organized around implementation, client and policy gaps and constraints (Table 4).

**Table 4:** Implementation, client and policy constraints to achieving equity

Implementation Constraints	Client Constraints	Policy Constraints
1. Lack of quality comprehensive FP services, characterized by frequent stock outs, long distances to health facilities reflecting poor physical access of facilities, scope of services that does not meet the needs of marginalized populations including adolescents 2. Limited access to postpartum family planning (PPFP) for both home and facility deliveries 3. Lack of male involvement in supporting some women to take up FP 4. Weak inter-ministerial and partner coordination to provide FP to high burden and hard-to-reach populations including (refugees, young girls, islands and mountainous settings 5. The long-acting reversible contraception and permanent methods are not closer to clients 6. Ineffective supply and distribution chain of FP commodities 7. Lack of commodity and service delivery mapping to track the availability of commodities at the facility level 8. Inadequate number of skilled providers and poor attitudes which limits access to wide range of methods 9. Inadequate funding for equitable family planning 10. Generic FP programs without considering the needs of underserved populations 11. Uneven distribution of FP programs and partners	1. The socio-cultural factors-myths and misconceptions, religious values and gender inequality in rural communities 2. Limited contraceptive information targeting the young people, rural women and men 3. The high client out of pocket payments in the private sector and high cost of LARC hinder the rural poor from accessing FP services	1. The National and health sector development plan II and other FP policy documents have less focus on equity 2. There are no well-designed sector-specific policies and programs on gender to facilitate equitable access to SRH information and services 3. Inequities in FP use have received little national acknowledgement and attention from health policy-makers 4. Lack of multi-sectoral approach to implement the National Adolescent Health Policy Action Plan 5. Policies and plans are not effectively implemented to address 6. Limited understanding of FP national policies by the implementers

#### Implementation constraints to achieving equity

The implementation constraints identified in the reviewed documents included FP program related factors which hinder the delivery and equitable access to and use of FP services especially in rural areas, hard-to-reach settings including islands, and mountainous communities. These were included in 11/25 documents [24, 26–29, 32, 33, 39, 41–43]. Similarly, some of the key informants noted that the generic FP programs which do not consider equity in design and implementation, lack of a common understanding of equity among implementers and the uneven distribution of donors, partners in different parts of the country are hindering achieving equitable FP. One of the key informants said:

*“You find that some districts have more FP partners who end up duplicating services while others don’t have any single partners implementing FP. How shall we achieve equity in that instance? Some groups such as persons with disability, young people, rural and slum areas have no specific interventions focusing on them”* (KI-Development partner)

Almost all of the key informants and stakeholders highlighted data challenges including lack of data for specific populations such as the very young (10–14 years), people with disabilities (PWDs), data often not disaggregated, not timely and incomplete. Stakeholders also noted the bureaucracies involved in accessing data, limited capacity to analyse and use data inform equitable FP programming.

#### Clients’ constraints to achieving equity

These included client related factors such as socio-cultural norms and myths, poverty, and high costs of long term methods among others that hindered equitable use of FP services. These constraints were highlighted in more than half (12/25) of the documents reviewed [23, 24, 27, 31–35, 38–41]. Similarly, the key informants also reported lack of access to information especially in rural areas, myths about FP and negative side effects of FP, as key constraints hindering equitable FP access. One of the key informants at sub national level noted:

*“The peasant farmers in rural areas who are the majority also spend most of the time in garden, hence miss out on FP information on radios*” (KI-Sub National Level)

#### Policy constraints to achieving equity

These include policy related restrictions and hindrances to equitable FP service delivery and access. More than half 15/25 of the documents [23, 24, 26–29, 32, 33, 35, 36, 39, 40, 42, 43, 46] highlighted a number of policy gaps constraining achieving equitable FP. Most of these related to policy inadequacy and poor implementation. Some of the key informants also noted that a number of policies do not emphasize equity. They also reported the limited awareness of the FP policies by the implementers and lack of multisectoral approach in implementing FP policies, as policy constraints to achieving equity.

## Discussion

This paper documents outcomes of a review of 25 documents and stakeholder consultations on how equity in FP is conceptualized, assessed and the constraints to achieving equity in FP programs and policy in Uganda.

Our findings show lack of a common understanding of equity among the different stakeholders including donors, policy makers and FP program implementers. The definitions varied across program and policy documents, usually focusing on only one dimension of equity. Most definitions of equity in Ugandan documents were not aligned to the FP2030 vision which indicates that each person has the same right and access to quality family planning, regardless of their geography, socioeconomic status, gender, or culture [12]. The varying definitions of equity were similarly reported by studies conducted in Tanzania [47], Rwanda [48], Burundi [49], Pakistan [50] and Cambodia [51], where equity was mainly defined basing on economic dimension and use of contraceptives as the main outcome [52–54]. To achieve the FP2030 commitments, there is need for a common understanding and alignment of the definition of equity within the FP2030 Agenda. A common understanding of equity is a pre requisite for a shared practice and implementation among actors [55]. Additionally, the current definitions deny policy makers and program implementers the chance to explicitly measure and target the disparities in desired fertility across different dimensions such as socio demographics and geographical location. Hence, policies and programs need to adopt a broad and common definition to consistently measure and track progress. Having common definition of equity is important for designing policies and programs with equity as focus and also guide implementation of equitable FP.

Equity was interchangeably used with equality among Uganda stakeholders. Whitehead (1992), noted that inequality could be the difference in health among different groups of people whereas inequity includes those differences which are avoidable, unnecessary and unjust [14]. Similar observations were noted in a review by Espinoza (2007) where equity and equality was defined interchangeably [56]. Conceptualizing equity as equality poses a risk of FP programs not being responsive to needs of the different groups of people, especially those left out like the rural, poor, very young and people living with disabilities. Therefore, there is need for a clear understanding among implementers that the concept of equal access to FP services does not necessarily address the underlying drivers of inequity, which need to be bridged.

It was noted that, there is lack of data for some populations such as for people with disabilities (PWDs), data is not always disaggregated (average estimates are used) to measure equity, which was reported to be hindering equitable FP programming. Similarly, a global health equity impact assessment noted the lack of data as one of the key hindrances of addressing equity in health programs [55]. Using average national estimates of family planning indicators mask important disparities in access and utilization of FP services across the different equity dimensions [57]. Therefore, it is critical that FP outcome data is collected capturing equity dimensions, stratified analyses and disaggregated by social, economic, geographical, and empowerment dimensions. The indicators should also be expanded to capture other groups such as the very young (10–14 years) and people living with disabilities, because often these are left out in programming and yet these for instance the young are faced with high unintended pregnancies and the associated consequences. Few studies/programs addressed equity in a multidimensional way. Most studies in this review focused mainly on geographical and wealth dimension for measuring equity as previously noted by Hardee et al., (2019) [53]. This limits understanding of the FP needs of the different population groups (Including the young 10–14 years, people with disabilities and other vulnerable populations) and sub national contexts. Therefore, in order to monitor these multi-dimensional disparities and target interventions to the underserved populations, there is need for stratified analysis of program data capturing all the equity dimensions including social, economic and environmental dimensions.

Regarding the FP outcomes used to measure equity, modern contraceptive prevalence rate was the most commonly used outcome for assessing equity. Relying on contraceptive use alone without factoring in the need for FP may be erroneous in identifying FP inequities [53, 57]. Analyses of inequity in FP, neglected the range of programmatic components that affect use, such as access to information and services, and good quality of care. This was similar to studies conducted in Tanzania [47], Rwanda [48], Burundi [49], Pakistan [50] and Cambodia [51], where equity was measured by use of contraceptives as the main outcome. There is need to broaden the outcomes when assessing equity to include unmet need for FP, fertility desires, demand satisfied, among others.

Most FP programs are designed targeting the general population without considering and engaging the underserved populations such as the young people in the intervention design who have specific needs and challenges [49]. There is need to determine the specific needs of the underserved populations and engage them in intervention designs so that FP services are targeted and no one is left behind. The uneven distribution of organizations supporting FP programs in the country exacerbates inequity and should be harmonized to ensure that some regions and districts are not left behind.

Other key implementation constraints of achieving equity included poor quality and lack of comprehensive FP services and method mix especially in rural areas. Thus, efforts are needed to improve the quality of FP services in rural settings. Interventions using community health workers, self-care approaches through the private drug shops, and subsidizing of FP commodities could be more effective in reaching the rural poor [58]. However, there is need to invest in quality assurance and monitoring to ensure that such improvements are standardized, routinized, and sustained even in the rural areas to improve FP outcomes.

The high cost of long-term contraceptives due to out-of-pocket payment was a constraint to achieving equity in FP especially among the poor. Removing the financial barriers to accessing and using FP services is much needed [59]. Research is also needed to better understand the best financing mechanisms and whether the introduction of a national health insurance scheme in Uganda will removal financial barriers to access and use of FP especially among the poor. Additionally, the socio-cultural norms, myths and misconceptions remain a huge barrier to use of FP especially in rural and hard to reach settings. This is coupled with limited access to accurate FP information. Similar observations have been reported in other studies in Uganda and in most African countries [59, 60]. Therefore, there is need to avail appropriate messages to demystify the myths and misconceptions of FP among communities. Ineffective and poor implementation of policies was also noted among key constraints of achieving equity. The national policies had no specific objectives on how to reach the underserved population. There is need to adopt the concept of equity in all FP policies, with a shared definition across sectors to ensure that policies promote equitable FP programming and access especially among those in most need.

### Limitations and strengths

Triaging of articles was based on title and abstract, which could have left out some relevant articles that missed equity issues in the title and abstracts. This search was also limited to English online documents published between 2010 and 2020 from Uganda and could have missed out some research articles. Therefore, these findings apply to Ugandan settings. However, we used a broad search strategy to ensure that critical articles are captured and reviewed. We also contacted key resource persons to access additional reports which the online search could have missed and the findings in this paper were augmented by insights from key informants and consultative workshops.

## Conclusion

This paper shows inconsistency in the definition and measurement of equity in FP among key informants and across policy and program documents in Uganda. Single dimension use for assessing equity was common, mostly focusing on economic (wealth) and geography (rural/rural), while mCPR, unmet need and demand satisfied were the commonest outcomes.

Multidimensional assessments and measurements of equity should be adopted to monitor inequities and target FP interventions to the underserved populations. There is need for stratified/disaggregated analyses of program data to precisely determine inequities in access to and use of FP in order to inform programming, policy and resource allocation. Addressing the implementation, client and policy constraints will be critical to achieving equity.

## Data Availability

The Google data extraction excel data sheet and notes from the key informants are available on reasonable request from the corresponding author.

## Abbreviations

BCC: Behavior change communication
CIP: Costed Implementation Plan
FP: Family planning
HIPs: High impact practices
mCPR: Modern contraceptive Prevalence rate
MOH: Ministry of Health
PPFP: Postpartum Family planning
PWDs: People with disabilities
UNFPA: United Nations Population Fund
USAID: United States Agency for international Development
WHO: World Health Organization
